# Accelerator trial series in *Pinus radiata* stands in New Zealand: Trial establishment, site description and initial soil, forest floor and tree data

**DOI:** 10.1016/j.dib.2023.108991

**Published:** 2023-02-16

**Authors:** Simeon J Smaill, Loretta G Garrett, Sarah L Addison

**Affiliations:** a Scion, PO Box 29237, Riccarton, Christchurch 8440, New Zealand; b Scion, Private Bag 3020, Rotorua 3046, New Zealand

**Keywords:** Planted forest, Productivity, Long-term trial, Sustainability

## Abstract

Interest in establishing biological-based economies has created increasing and rapidly moving demand for wood and fibre from production forests. Meeting the global demand for timber supply will require investment and development across all components of the supply chain but will ultimately rely on the ability of the forestry sector to increase productivity without compromising the sustainability of plantation management. To address this issue in the context of New Zealand forestry, a trial series was established from 2015 to 2018 to accelerate plantation forest growth by exploring current and future limitations to timber productivity, then altering management practices to overcome these limits. The six sites in this Accelerator trial series were planted with a mix of 12 different types of *Pinus radiata* D. Don stock expressing various traits related to tree growth, health and wood quality. The planting stock included ten clones, a hybrid and a seed lot representing a widely planted tree stock used throughout New Zealand. At each trial site a range of treatments were applied, including a control. The treatments were designed to address the specific current and predicted limitations to productivity at each location, with consideration for environmental sustainability and impacts on wood quality. Additional site-specific treatments will be implemented across the approximately 30-year life span of each trial. Here we present data describing both the pre-harvest and time zero state of at each trial site. These data provide a baseline that will enable treatment responses to be holistically understood as the trial series matures. This comparison will determine if current tree productivity has been enhanced, and if improvements in site characteristics may also benefit future rotations. The Accelerator trials represent an ambitious research goal that will take planted forest productivity to a new level of enhanced long-term forest productivity without compromising the sustainable management of future forests.

**Specifications Table**

**Table:** 

Subject	Forestry
Specific subject area	Sustainably enhancing planted forest productivity through site improvements for current and future site limitations.
Type of data	Tables and images.
How the data were acquired	Field data collection, measurement and laboratory analysis of soil and forest floor. At five sites sample plot locations acquired with high resolution GPS, which were then used to derive elevation, slope, aspect and climate from spatial topographic layers created by LiDar. At one site slope and aspect were not derived from LiDar, and were instead measured on-site.
Data format	Raw and analysed.
Description of data collection	The data were collected from six long-term Accelerator trial sites in New Zealand. Pre-harvest at sites where the opportunity was present and time-zero data was collected to provide a baseline for the beginning of the Accelerator trial series.
Data source location	Scion, New Zealand. Location of the six trials, latitude and longitude, are in data tables.
Data accessibility	Data sets are provided with this article and available in the Figshare repository: https://doi.org/10.6084/m9.figshare.21273114.v1
Related research article	N/A

## Value of the Data

• The data provides baseline data to New Zealand's Accelerator trial series upon which all other collected data from these trials will be compared to.
• The data can be used to determine pre-harvest biomass, carbon and nutrient pools at each site at a plot level, fully characterising the starting conditions prior to any treatment.
• The data will benefit New Zealand and global forestry science targeted towards a) sustainable enhancement of forest productivity, and b) continuously improving best management practices for the New Zealand and global forestry industry.
• The data can also be used to increase knowledge of the variation in planted forest soil properties in relation to the productivity limitations, providing a framework for additional trials and experiments addressing limitations associated with site specific factors.
• The data can also be used to support the establishment of experimental gradients in specific site factors to explore their impact on short- and long-term (∼30 years) forest productivity.

## 1 Objective

The dataset was generated to allow the impact of various management treatments on forest and forest soil properties to be determined by comparison to pre-treatment conditions. The range of soil properties detailed in the dataset is intended to allow holistic assessment of the treatment outcomes over time, indicating their effects on both the productivity and sustainability of forestry operations. This latter factor is of considerable importance given the relatively long life span of planted forests.

## 2 Data Description

**Fig. 1:**
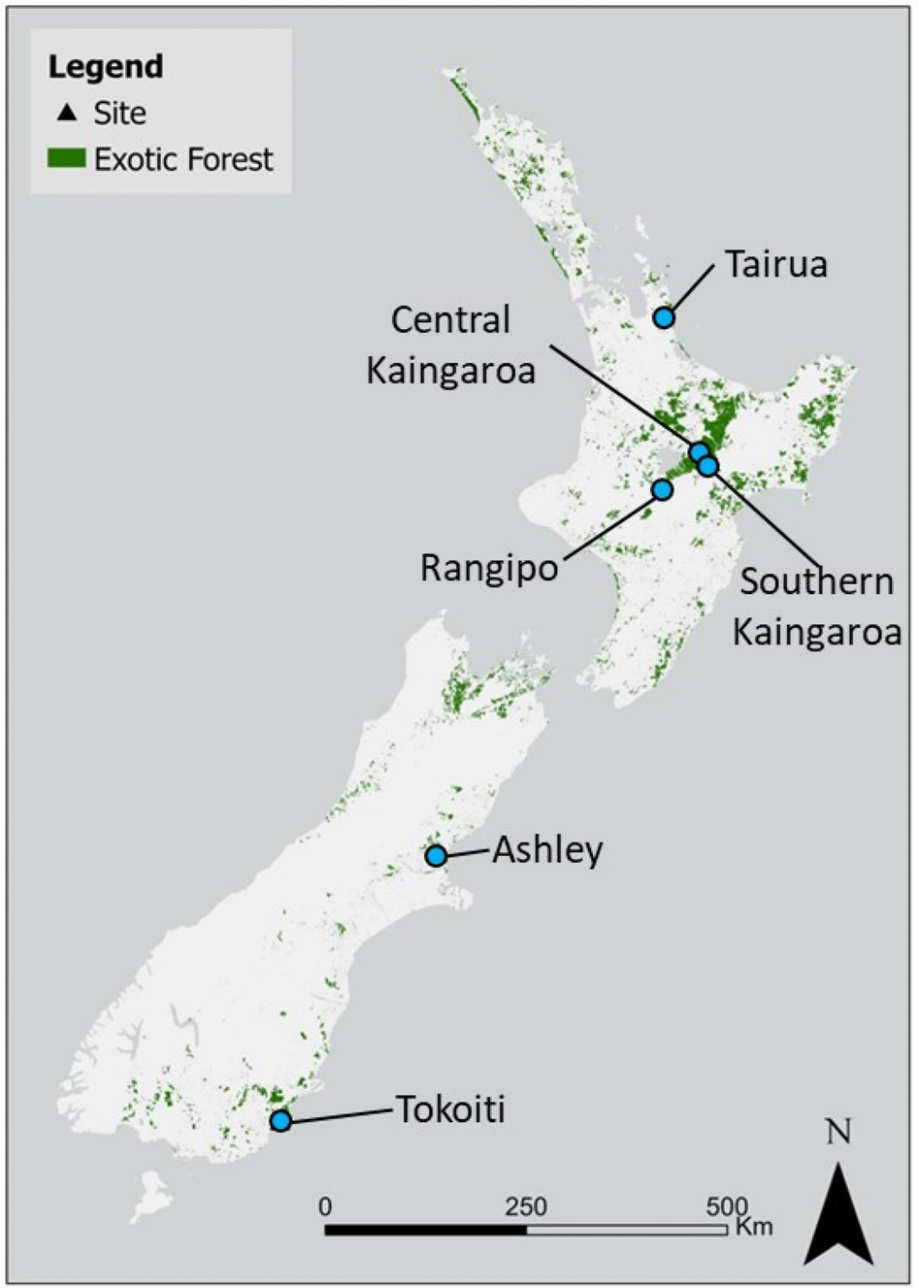
Location of the six Accelerator trials within New Zealand.

The dataset contains raw and summarised data collected from six Accelerator trials in New Zealand *Pinus radiata* forests. Table 1 provides a description for each of the trial sites, Table 2 lists the traits of the tree material planted at the sites, Table 3 indicates what planting material was planted at each of the trial sites, and Fig. 1 shows the location of the six Accelerator trials within New Zealand.

**Table 1:** Site description and trial design.

Variable	Site					
Trial identifier	FR556/1	FR556/2	FR556/3	FR556/4	FR556/5	FR556/6
Site	Southern Kaingaroa	Rangipo	Central Kaingaroa	Ashley	Tairua	Tokoiti
Latitude (°)	−38.80	−39.09	−38.53	−43.22	−37.16	−46.20
Longitude (°)	176.50	175.83	176.55	172.56	175.79	170.00
Elevation (m above sea level)	757	544	458	224	179	128
Mean annual temperature ( °C) [5]	10.0	10.7	11.3	11.3	14.1	10.2
Annual rainfall (mm) [5]	1463	2038	1201	799	2049	807
Slope (degrees)	4	4	4	16	11	6
Soil parent material	Volcanic flow deposits from the Taupo volcanic centre (1860 ± 100 Before Present (BP))	Volcanic air fall deposits from the Taupo volcanic centre (1860 ± 100BP)	Volcanic air fall deposits from the Taupo volcanic centre (1860 ± 100BP)	Interbedded grey-wacke gravels, silts, and clays	Volcanic air fall deposits over weathered andesite lava or ignimbrite deposits	Loess deposits
Soil texture [9]	Moderately to very gravelling loamy sand and sand	Sandy loam and loamy sand	Sandy loam, loamy sand and sand	Clay loam over a gravelly sandy clay loam	Sandy loam and loamy sand over a clay loam	Silt loam
Soil drainage [9]	Well drained	Well drained	Well drained	Well to imperfectly drained	Well to imperfectly drained	Imperfectly drained
Rooting barrier [9]	No significant barrier within 1 m	No significant barrier within 1 m	No significant barrier within 1 m	No significant barrier within 1 m	No significant barrier within 1 m	Soil horizon with fragipan properties between 65 – 88 cm (average = 75 cm)
New Zealand Soil Classifcation [6]	Immature Orthic Pumice	Typic Orthic Pumice	Immature Orthic Pumice	Typic Immature Pallic and Mottled Immature Pallic	Vitric Orthic Allophanic soil where the volcanic air fall deposits are thick enough to an Allophanic or Mottled Orthic Pumice and Acidic Allophanic Brown Soil	Mottled Fragic Pallic
Planted (year)	2015	2016	2016	2017	2017	2018
Trial area (ha)	9.6 ha	13.4 ha	5.9 ha	10.2	7.0	9.9
Target tree stocking at establishment (stems ha^−1^)	1000	833 and 1282	1000	1000	1282	1000
Mean plot area (ha)	1.1	1.4 ha (833 stems ha^−1^)				
0.8 ha (1282 stems ha^−1^)	1.0	1.1	0.8	1.0		
Forest rotation number at start of trial	3	1	3	3	3	2
Previous crop	*P. radiata*	Pasture	*P. radiata*	*P. radiata*	*P. radiata*	*P. radiata*
Previous crop end of rotation age (yr)	31	n/a	30	27	28	30
Previous crop end of rotation mean basal area (m^2^ ha^−1^)	43.3	n/a	51.4	70.2	68.2	68.0
Previous crop end of rotation mean top height (m)	36.3	n/a	42.5	33.2	41.4	37.3
Previous crop end of rotation mean annual increment (m^3^ year^−1^)	16.85	n/a	24.00	28.84	33.25	26.70
Previous crop end of rotation mean tree stocking (stems ha^−1^)	235	n/a	273	459	424	271

**Table 2:** List of tree planting material traits.

	Stock Traits					
Planting stock identity	Enhanced DBH	Enhanced modulus of elasticity	Enhanced density	Reduced density	Dothistroma resistant	Drought tolerant
Clone A	✓	✓	✓		✓	✓
Clone B	✓	✓		✓		
Clone C	✓	✓	✓			✓
Clone D	✓	✓	✓			
Clone E	✓	✓		✓		
Clone F					✓	✓
Clone G	✓	✓		✓		
Clone H	✓	✓	✓		✓	
Clone J	✓	✓		✓		
Clone K	✓	✓	✓			
Clone L	✓					
Clone M	✓	✓	✓		✓	
Clone N	✓	✓		✓		
Clone O	✓					
Clone P	✓	✓				
A x C hybrid						✓
GF19

**Table 3:** list of tree planting material by site.

	Site					
Planting stock identity	FR556/1	FR556/2	FR556/3	FR556/4	FR556/5	FR556/6
	Southern Kaingaroa	Rangipo	Central Kaingaroa	Ashley	Tairua	Tokoiti
Clone A	✓	✓	✓	✓	✓	✓
Clone B	✓	✓	✓	✓	✓	✓
Clone C	✓	✓	✓	✓	✓	
Clone D	✓	✓	✓			
Clone E	✓	✓	✓			
Clone F	✓	✓	✓			
Clone G	✓	✓	✓	✓	✓	
Clone H	✓			✓	✓	✓
Clone J	✓	✓	✓	✓	✓	✓
Clone K	✓	✓	✓	✓	✓	✓
Clone L				✓	✓	✓
Clone M		✓	✓	✓	✓	✓
Clone N						✓
Clone O				✓	✓	✓
Clone P						✓
A x C hybrid	✓	✓	✓	✓	✓	✓
GF19	✓	✓	✓	✓	✓	✓

The raw data on initial soil, forest floor and trial details for the accelerator trial series (FR556) in *Pinus radiata* stands in New Zealand are stored in Figshare [1].

## 3 Experimental Design, Materials and Methods

### 3.1 Site Selection and Description

Candidate locations for site establishment were evaluated based on three criteria:

• The locations were within existing production forests, or in locations where significant afforestation efforts were planned (Fig. 1), allowing all trial results to have wider relevance to forest management practice
• Productivity gap analysis [2,3] at the location showed that current productivity was sub-optimal, indicating there is an opportunity to increase productivity through methods identified by the gap analysis at that site, and other similar locations.
• The location was known to possess a specific property or limitation that can be treated in an experimental framework to improve productivity at that location, and other similar locations.

A total of six locations were selected and a trial site installed (Table 1). The principal limitations to productivity for each site were:

• FR556/1 Southern Kaingaroa – available nutrients, specifically nitrogen.
• FR556/2 Rangipo – a fertile site where the boundaries of silviculture, tree stocking, can be increased.
• FR556/3 Central Kaingaroa – soil organic carbon and nitrogen.
• FR556/4 Ashely – climate is dry, specifically soil moisture is limited.
• FR556/5 Tairua – available nutrient specifically phosphorus.
• FR556/6 Tokoiti – future climate will be dry within the current forest rotation [4] and soil moisture will become limited.

### 3.2 Trial Design

Each trial site was established with replicated treatment blocks, varying with the number of treatments applied at that site. Within each plot there are 12 sub-plots planted with different radiata pine planting stock. Each sub-plot contains 49 measurement trees on a seven tree by seven tree grid, with a one tree buffer surrounding the measurement trees producing a nine by nine grid. The exception to this was the Tairua site, at which each sub-plot contained 48 measurement trees on a six by eight grid. Blocking was used where necessary to account for spatial variation and each plot and sub-plot position was selected randomly.

At each site sub-plot center location, slope, aspect, elevation and PSP identity were recorded [1]. High resolution LiDar data was captured at each site to create a topographical spatial layer, allowing elevation, slope and aspect to be derived for each sub-plot. The exception to this process was Tokoiti; at this site slope and aspect were measured on site using a hand-held slope meter and compass, while for seven sub-plots elevation was not derived due to missing LiDar data.

Accelerator trials sub-plots were planted with one of a selection of twelve radiata pine stock, including ten clones, a hybrid (*Pinus attenuata x P. radiata* var. *cedrosensis*, hereafter A x C) and a seed lot representing conventional planting stock (GF19; Growth and form on the GF rating scale [7]) used throughout New Zealand. Productivity and wood quality were key factors in clone selection. Selection for inclusion in the trials was based on the following traits [8]: enhanced rates of diameter growth at breast height (DBH; 1.4 m stem height); enhanced modulus of elasticity (wood stiffness, an accepted indicator of wood strength); degree of wood density; dothistroma (*Dothistroma septosporum*) needle blight resistance; and drought tolerance (Table 2). All traits were defined with reference to conventional GF19. There is some stock variability between sites due to different stock supply from year to year, however, the stock traits were able to be kept consistent (Table 2 and Table 3). Planting stock was raised following consistent nursery practices (e.g. fertiliser and herbicide applications) for all stock across all years.

Treatment selection and implementation at each site was targeted to address the research goal: *removing site specific limitations to forest productivity using multiple treatments that are guided by state-of-the-art science to improve the site itself, and not just the current crop.*

Time zero treatments were applied at Rangipo and Tokoiti sites. At the Rangipo site a pre-planting treatment was applied by rip mounding half of the site, following standard establishment practice, and half of the site was left uncultivated. Furthermore, at planting two stocking rates were used: 833 stems per hectare (sph) and 1282 sph. The greater stocking rate was established to explore site carrying capacity as it was considered that the conventional stocking rate may not fully utilise the soil resources at the site. At the Tokoiti site treatments to increase the soil organic carbon content and soil rooting depth were applied to enhance water storage capacity. Treatments were: Whole tree harvesting followed by windrowing; whole tree harvesting followed by grinding of the harvest residue and cultivation of the mulch into the soil to a depth of 500 mm; stem only tree harvesting followed by grinding of the harvest residue and cultivation of the mulch into the soil to a depth of 500 mm.

### 3.3 Pre-Harvest Sampling

Pre-harvest data was collected from three of the Accelerator sites where there was an opportunity to measure forest floor and soil data.

### 3.4 PSP Installation

Three 0.04 ha PSP's were installed per site, either adjacent to the trial area (FR556/1, FR556/3) or within the trial area (FR556/6) just prior to harvest. Slope was measured using a handheld slope meter for each PSP; all PSP were flat (0° slope) for FR556/1 and FR556/3. Slope was 8, 8 and 5° for PSP FR556/6/201, FR556/6/202, FR556/6/203, respectively.

### 3.5 Forest floor and mineral soil

Forest floor material, fresh litter (L) and partly and well decomposed litter (FH) (<10 cm diameter) were sampled from 12 locations per plot. The 12 sampling points were positioned at one random sampling point per plot quarter and at each point three samples were collected 1 m apart. The L horizon was sampled using a 0.1 m^2^ sampling square. The FH horizon was sampled using a 98 mm internal diameter stainless steel ring. The L and FH samples were bulked separately for each plot. Mineral soil samples for chemical analysis were collected in the following increments down to 30 cm depth (0–5, 0–10, 10–20, 20–30 cm depth) using a 25 mm diameter stainless steel Hoffer tube sampler from 30 points in a grid pattern over each plot and bulked by plot. At Tokoiti this protocol was varied, with 20 sampling points per plot, collected from sampling depths of 0–10, 10–20 and 20–30 cm depth. Soil bulk density samples were collected in increments down to 30 cm depth (0–10, 10–20, 20–30 cm depth) from two randomly selected points per plot using a 98 mm internal diameter stainless steel ring. The data from each plot and depth increment were summed to make one bulk density value per depth increment for each plot.

### 3.6 Time Zero Soil Sampling

Time zero soil samples were collected within 1–2 months after planting the Accelerator trials. Soil chemistry samples, 0–10 cm depth, were collected within the tree measurement area of all sub-plots using a 25 mm diameter stainless steel Hoffer tube sampler. At Southern Kaingaroa, Central Kaingaroa and Rangipo the 0–10 cm samples were collected from both within the tree row and on the tree site preparation mound on a grid pattern and bulked per sub-plot. At Southern Kaingaroa, Central Kaingaroa 32 sample points between tree rows and at Rangipo 30 samples between tree rows were collected within the measurement sub-plot area and for each of the inner sub-plot 5 × 5 trees two samples were collected from each tree position on the soil mound (50 bulked samples). At Ashely, Tairua, and Tokoiti 30 samples were also collected between tree rows. Deeper soil samples were collected from the GF19 sub-plots at all sites; these samples were collected from positions between the tree rows from10–20 and 20–30 cm depth.

Soil bulk density samples were collected to depth (0–10, 10–20, 20–30, 30–50, 50–100 cm) from within the tree buffer area at one corner of each of the GF19 sub-plots using a 98 mm internal diameter stainless steel ring. Deep soil chemistry samples, 30–50 and 50–100 cm, utilised the soil bulk density samples collected from the GF19 sub-plots. Another set of soil bulk density samples (0–10, 10–20, 20–30 cm) were collected from the GF19 sub-plots from a random sample point within the sub-plot tree buffer. The data from these GF19 sub-plot bulk density samples were summed to make one bulk density value for each sub-plot. An additional soil bulk density sample, 0–10 cm depth, was collected from all sub-plots planted with C15 and C43 planting stock from a random sample point within the sub-plot tree buffer. The 0–10 cm bulk density values from GF19, C15 and C43 were averaged by plot to give one bulk density value for each plot.

At each deep soil sampling point (GF19 sub-plots) a soil profile photo was taken and a basic soil profile description was undertaken capturing data on soil horizons including; horizon notation, depth, gravel volume% and description, mottle%, drainage, and for selected soil profiles; texture, colour, soil parent material, and reactive aluminium test using methods described in Milne et al. [9]. Soil classification for each deep soil point were undertaken using the New Zealand Soil Classification system [6] (Table 1).

### 3.7 Sample Preparation and Testing

#### 3.7.1 Forest Floor

Forest floor (L and FH) samples were oven dried at 70 °C to constant weight, weighed and then ground in a Wiley mill to pass a 1 mm screen. Samples were tested for total carbon and total nitrogen using a LECO FPS-21,000 CNS thermal combustion furnace. A range of total elements (calcium, potassium, magnesium, sodium, aluminium, boron, copper, iron, manganese, and zinc) were determined following microwave assisted dry digestion, then 1:10 dilution with type 1 water and analysed by an Inductively Coupled Plasma Spectrometry (ICP-MS). Loss on ignition was determined at 525 °C. All results are reported on an oven-dry (104 °C) basis.

#### 3.7.2 Soil

Mineral soil chemistry samples were air-dried (<40 °C) and then sieved to retain the <2 mm and >2 mm mineral soil fractions and archived. The soil bulk density sample mineral soil fractions (<2 mm and >2 mm) and >2 mm organic matter fraction were oven dried at 104 °C and weighed. Pre-harvest and time zero soil samples collected from sub-plots targeted for deep soil sampling (0–10, 10–20, 20–30, 30–50, 50–100 cm) were selected for a range of chemical analysis. The selected soil samples (<2 mm fraction) were tested, where possible for all of the following; pH measured by 1:2.5 soil/water suspension; total carbon and total nitrogen using a LECO FPS-21,000 CNS thermal combustion furnace; available phosphorus using FIA colorimetry after sequential 1:10 Bray 2 (NH_4_F/HCl) extraction; available elements (aluminium, boron, calcium, copper, iron, magnesium, manganese, sodium, phosphorus, potassium, zinc) using ICP-MS (inductively coupled plasma mass spectrometry) after Mehlich 3 extraction; total phosphorus using FIA colorimetry after sulphuric acid digest; exchangeable cations (calcium, potassium, magnesium and sodium) using an ICP-MS after 1:50 (macro) NH_4_CH_3_COO leaching; inorganic and organic phosphorus using FIA after H_2_SO_4_ extraction and ashing. All results were reported on an oven-dry (104 °C) basis. Sulphate-S was measured by IC (Ion Chromatography) after 0.02 M potassium phosphate extraction and reported on an air-dry basis of 35–40 °C. Time zero soil samples were additionally tested for elemental totals; aluminium, arsenic, boron, barium, calcium, cadmium, cobalt, chromium, copper, iron, potassium, magnesium, manganese, sodium, nickel, phosphorus, lead, sulphur, selenium, strontium, thallium, uranium, vanadium, and zinc (Al, As, B, Ba, Ca, Cd, Co, Cr, Cu, Fe, K, Mg, Mn, Na, Ni, P, Pb, S, Se, Sr, Tl, U, V, and Zn) as measured by ICP-MS (inductively coupled plasma mass spectrometry) using a modified USEPA digestion method 3050B to include a reverse aqua regia digest of 1 HCl: 3 HNO_3_ to limit Cl^−^ interference. Results are reported on an oven dry basis of 50 °C.

Diffuse reflectance MIR spectra were acquired for the pre-harvest and time zero sub-set of soil samples selected above for chemical analysis using methods described in Garrett et al., [10]. In summary, a representative 10 g soil sub-sample of the archive soil (<2 mm soil fraction) was ground for 180 s in a 45 ml zirconia ceramic grinding vial containing two 12.7 mm zirconia ceramic balls using a Spex800D mixer mill. Diffuse reflectance MIR spectra were acquired for all samples, using a Bruker Invenio-S Fourier transform infrared (FTIR) spectrometer with a Bruker HTS-XT (High Throughput Screening Extension) microplate reader fitted with a liquid nitrogen cooled mercury cadmium telluride detector. The system was purged with CO_2_-free dry air using a Peak Scientific PG14L generator. Four replicate samples were scanned, and sample spectra were acquired in diffuse reflectance mode with 50 co-added scans from 8000 to 400 cm^−1^ at 4 cm^−1^ resolution. Spectra were pre-processed using Bruker OPUS 8.2 software applying the make scalar compatible and cut functions to truncate the wavenumber range to 4000 to 600 cm^−1^.

#### 3.7.3 Soil Microbial Properties

A sub-sample of the field fresh soil from the soil chemistry sample was sieved to <2 mm and ∼1 g was archived for future soil DNA testing in a −80 °C freezer, and the remaining sieved soil was assessed using the MicroResp™ platform as described below

The ecophysiological status of soils from all sites were assessed using the MicroResp™ system [11]. Briefly, freshly collected and sieved (< 2 mm) soils were added to each well in a 96-format deep-well plate. Each well contained known amount of soil (ranging from 300 to 600 mg), pre-adjusted to 40% water holding capacity. Plates were sealed and preincubated in the dark for 5 days at 20 °C. The responses to addition of 14 carbon substrates, selected for ecological relevance to the soil microbial community, were assessed [11]. Substrates added were: d-galactose, d-glucose, d-xylose, α-cyclodextran (all delivered to produce a final 30 mg *g*^−1^ dry soil final concentration), L‑serine, glycine, Llysine, DL-aspartic acid, l-arginine, l-glutamic acid, l-threonine, 4-hydroxybenzoic acid, α-ketobutyric acid, and 2-phenylethylamine hydroxychloride (all delivered to produce a final 15 mg *g*^−1^ dry soil final concentration). Carbon sources were individually delivered in solution into individual soil samples, raising the soil water holding capacity in all wells to 60%. Once the carbon sources were added, each plate was paired to a detection plate using a silicon gasket [11]. Detection plates consisted of an agarose gel with a pH indicator dye, providing a colorimetric change following absorption of CO_2_. The colorimetric change, based on CO_2_ production by soil microorganisms, was determined by comparing readings of the detection plate immediately before incubation and 5 h after incubation at 20 °C using a microtitre plate reader at 590 nm. Results were standardised to the control (water addition only) wells.

## Ethics Statement

This work that did not involve studies with animals and humans, and no statement is required.

## Data Availability

The raw data on initial soil, forest floor, tree, and trial details for the accelerator trial series (FR556) in *Pinus radiata* stands in New Zealand are in Figshare [1]

## CRediT authorship contribution statement

**Simeon J Smaill:** Conceptualization, Methodology, Investigation, Data curation, Writing – original draft, Writing – review & editing, Supervision. **Loretta G Garrett:** Conceptualization, Methodology, Investigation, Data curation, Writing – original draft, Writing – review & editing. **Sarah L Addison:** Methodology, Investigation, Data curation, Writing – review & editing.

## Declaration of Competing Interest

The authors declare that they have no known competing financial interests or personal relationships that could have appeared to influence the work reported in this paper.

The authors declare the following financial interests/personal relationships which may be considered as potential competing interests:

Please declare any financial interests/personal relationships which may be considered as potential competing interests here.

## Data Availability

Raw data on initial soil, forest floor, and trial details for the accelerator trial series (FR556) in Pinus radiata stands in New (Original data) (Figshare).
